# A haplotype-based normalization technique for the analysis and detection of allele specific expression

**DOI:** 10.1186/s12859-016-1238-8

**Published:** 2016-09-13

**Authors:** Alan Hodgkinson, Jean-Christophe Grenier, Elias Gbeha, Philip Awadalla

**Affiliations:** 1 CHU Sainte Justine Research Centre, Department of Pediatrics, Faculty of Medicine, Universite de Montreal, 3175 Chemin de la Cote Sainte Catherine, Montreal, QC Canada; 2 Ontario Institute of Cancer Research, Toronto, ON Canada; 3 Department of Medical and Molecular Genetics, Guy’s Hospital, King’s College London, London, SE1 9RT UK; 4 Department of Molecular Genetics, University of Toronto, Toronto, ON Canada

**Keywords:** Allele specific expression, RNA sequencing, Normalization

## Abstract

**Background:**

Allele specific expression (ASE) has become an important phenotype, being utilized for the detection of *cis*-regulatory variation, nonsense mediated decay and imprinting in the personal genome, and has been used to both identify disease loci and consider the penetrance of damaging alleles. The detection of ASE using high throughput technologies relies on aligning short-read sequencing data, a process that has inherent biases, and there is still a need to develop fast and accurate methods to detect ASE given the unprecedented growth of sequencing information in big data projects.

**Results:**

Here, we present a new approach to normalize RNA sequencing data in order to call ASE events with high precision in a short time-frame. Using simulated datasets we find that our approach dramatically improves reference allele quantification at heterozygous sites versus default mapping methods and also performs well compared to existing techniques for ASE detection, such as filtering methods and mapping to parental genomes, without the need for complex and time consuming manipulation. Finally, by sequencing the exomes and transcriptomes of 96 well-phenotyped individuals of the CARTaGENE cohort, we characterise the levels of ASE across individuals and find a significant association between the proportion of sites undergoing ASE within the genome and smoking.

**Conclusions:**

The correct treatment and analysis of RNA sequencing data is vital to control for mapping biases and detect genuine ASE signals. By normalising RNA sequencing information after mapping, we show that this approach can be used to identify biologically relevant signals in personal genomes.

**Electronic supplementary material:**

The online version of this article (doi:10.1186/s12859-016-1238-8) contains supplementary material, which is available to authorized users.

## Background

Allele specific expression (ASE) is a phenomenon by which the expression of the two parental alleles is unbalanced. Over the past decade, the rise of high-throughput sequencing has facilitated the detection of ASE via RNA sequencing data [1–4], allowing for the detection of signals associated with *cis*-regulatory variation, loss of function alleles, imprinting and other important biological phenomenon [5–10]. ASE has also been implicated in human disease, either as a signal of variable gene expression or via the impact on the penetrance of damaging alleles [7, 11]. However, despite its potential use as a key indicator of biological processes, genuine ASE events can be difficult to detect without bias. Existing experimental approaches such as genotyping arrays or padlock probes still face many challenges [2], and although RNA sequencing can be used to identify events transcriptome wide, many factors can influence the detection of ASE from RNA sequencing data, including both technical and mapping issues [12, 13], which need to be corrected before reliable biological conclusions can be drawn. Perhaps the most accessible of these is sequence mapping biases, particularly since correction methods can be used to improve signals within the plethora of pre-existing datasets rather than requiring complete experimental re-design.

In order to detect ASE events from RNA sequencing data, a binomial test is often implemented to detect significant differences from the expected 50:50 ratio of alleles. This approach can be limited by coverage and power constraints, and consequently more advanced techniques are being developed [14, 15]. Prior to this, all current approaches rely on sequence read alignment and some form of correction procedure. Pre-existing mapping correction methods mostly fall into two camps: filtering potentially problematic sites [9, 13] and mapping separately to each parental genome [16–18], which is similar in concept to recoding the reference genome with standard ambiguous nucleotide codes prior to mapping [1]. The former approach is appealing since it is relatively simple to implement, however it runs the risk of removing problem areas of the genome that could still show biologically relevant signals if handled properly, and also potentially over-generalises effects by relying heavily on known biases and common SNPs in the genome, missing personal genome effects. Mapping to parental genomes, although appearing to be more accurate [16], requires not only assumptions about phasing and SNP calling from parental genomes, but also a large amount of complex manipulation and processing time; something not readily applicable to large high-throughput analysis and big data projects. Here, we propose a new approach that normalizes mapped RNA sequencing data by generating an assumed null dataset that can be used for correction of real data. We find that this approach leads to an improvement in reference allele quantification across a number of different mapping strategies and also compares favourably in either performance or processing time to alternative techniques when calling instances of ASE.

## Results and discussion

Typical identification of ASE from RNA sequencing data involves considering the ratio of reference and alternative alleles within sequencing reads that overlap heterozygous single nucleotide variants (SNVs), identifying sites that deviate significantly from the expected 50:50 ratio. However, mapping biases can lead to observed allele ratios in sequencing data that do not reflect the underlying biological state.

**Fig. 1:**
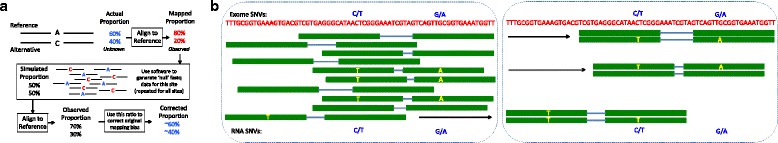
**a** Schematic for the normalization procedure. For a given heterozygous SNV the underlying proportion of reference and alternative alleles is unknown. After mapping, the proportion of reference/alternative alleles is observed, but may contain biases. To correct for this, a null dataset is generated for this site containing a 50:50 ratio of the two alleles (see panel **b**), and this data, together with null data from all other heterozygous sites is mapped using the same procedure as used for the original alignment. The observed proportion of mapped alleles from the null dataset is then used to correct the original data. **b** Generation of the null dataset. All reads and read pairs covering a heterozygous SNV are shown in the left hand panel. From these data, read pairs are randomly selected and the second haplotype is generated from known SNV data for the individual. In the right hand panel, three examples of this process are shown. At the top, the original read pair contains the reference allele at the SNV of interest (C/T), as well as the reference allele at a neighbouring SNV (G/A). The second haplotype is thus generated with the alternative alleles at both positions. In the middle, the original read pair contains two alternative alleles at the SNV sites, so an alternative read pair is generated with both reference alleles. At the bottom, the read pair contains the reference allele at the central SNV site, and what appears to be a sequencing error upstream at a site where no SNV has been identified. As such, a read pair is created with the sequencing error unchanged, and the alternative allele at the SNV position. This process is repeated for all read pairs to generate a null dataset with coverage of 4000X, and reads are converted into *fastq* format for remapping

In order to account for these biases we apply the following general approach (for more details see methods and Additional File 1): First, we map an RNA sequencing dataset to a reference genome. Second, we map corresponding exome sequencing data and call SNVs and indels. In practice, genomic SNVs and Indels can be obtained from DNA via any technology and coded in VCF format. Third, we generate a null high coverage RNA sequencing dataset that contains all SNVs and indels uncovered from the exome data, present at exact 50:50 ratios for the two parental alleles at all heterozygous sites. To do this, we sample read pairs from the original mapped RNA dataset and then recreate the two parental haplotypes in each case, repeating the process with replacement to generate a high coverage null dataset. Since the genetic background of variants is only important within reads for the simulation, variants do not need to be pre-phased. Fourth, we map the null sequencing dataset using the same software/pipeline as used in step one. In this way, any biases in the mapping procedure can be uncovered. Finally, we use the mapping biases observed in the mapped null dataset to normalize the original data (Fig. 1). A software package to generate a null high coverage dataset and to analyse results after mapping is available at https://github.com/AJHodgkinson/ASE.

### Simulations

To test the performance of our normalization approach, we generated simulated RNA sequencing data with gene expression levels and parental allelic ratios at heterozygous sites that matched real data. We then mapped these data using two commonly used aligners, Tophat2 [19] and STAR [20], the latter of which is the current gold-standard mapping tool suggested in the GATK [21] pipeline. In each case, we also varied some of the parameters used for mapping to consider which approach was most accurate. For Tophat2 we used both the default parameters (which, among other parameters, allows for a maximum of 2 mismatches per read), and also allowed for 5 mismatches per read, which is consistent with the default values used in other aligners. For STAR, we mapped the data using the default parameters and also after disabling soft-clipping of reads. For each simulation, the proportion of reads containing the reference allele at each heterozygous site was varied, generating a unique RNA sequencing dataset in each case and allowing for the full spectrum of biases to be explored.

**Fig. 2:**
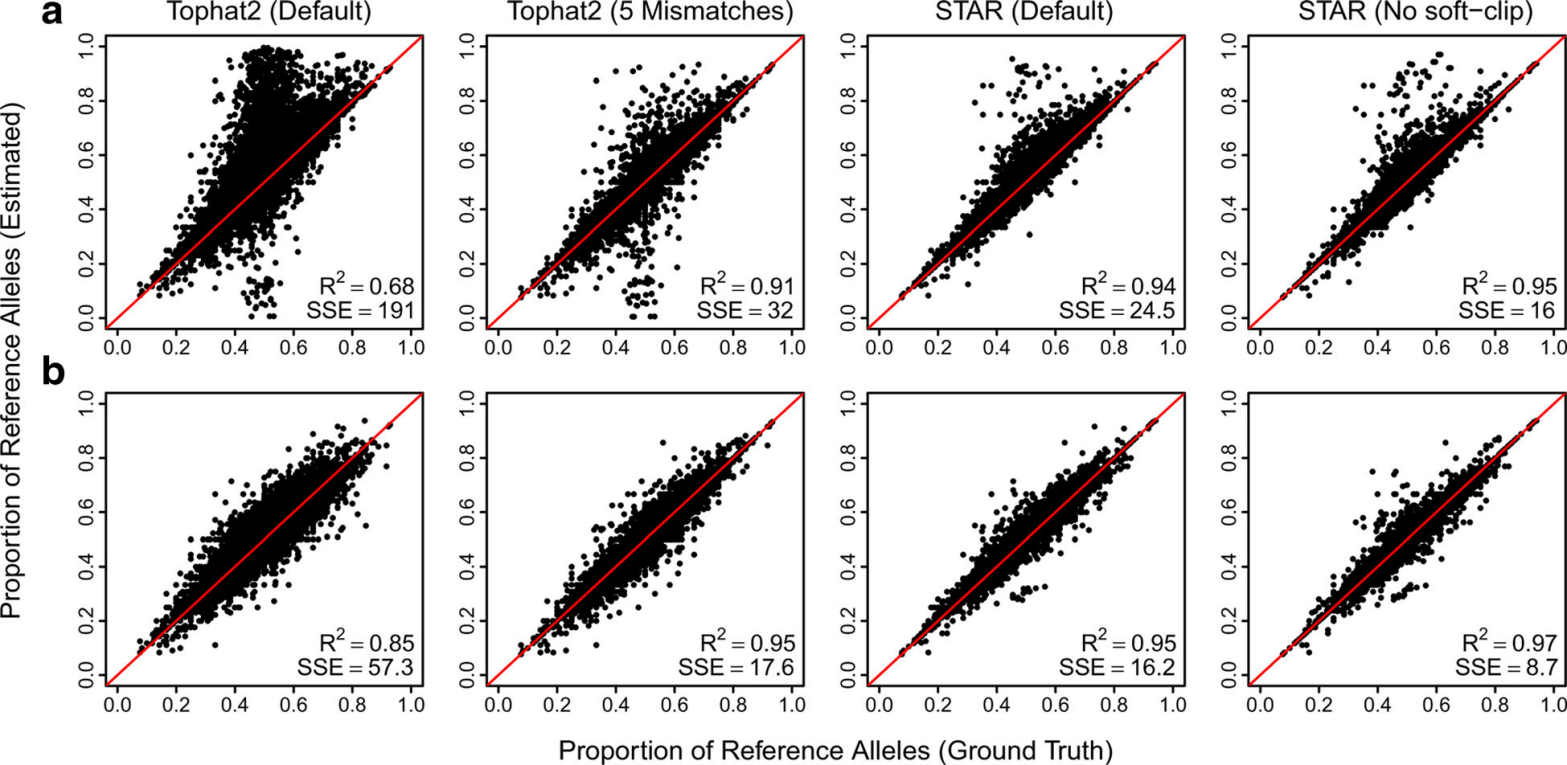
The proportion of reference alleles at heterozygous sites before and after normalization. Each plot shows the combined results from five simulated datasets, with the known reference proportion (ground truth) on the x-axis and the reference proportion obtained from aligning sequencing data (estimated) on the y-axis. **a** Shows the results obtained from initial mapping under four different approaches, and (**b**) shows the results of the same approaches after normalization. The sum of squared errors (SSE) is calculated around the red line (x = y), whereas R^2^ is obtained from analysing the correlation between the two variables

For each of the four mapping approaches, we generated five independent simulated datasets, mapped reads to the reference genome and compared observed parental allele proportions at heterozygous sites to the ground truth. Mapping with STAR after disabling soft clipping performed best (sum of squared differences between mapped and ground truth for proportion of reference alleles at heterozygous sites (SSE) = 16.04, R^2^ = 0.954 for correlation of data points), followed by default mapping with STAR (R^2^ = 0.943, SSE = 24.48), mapping with Tophat2 and allowing for 5 mismatches per read (R^2^ = 0.912, SSE = 32.01) and then default mapping with Tophat2 (R^2^ = 0.677, SSE = 191.3) (Fig. 2a). Importantly, the accuracy was significantly improved after applying our normalization approach in all cases (Wilcoxon signed-rank test to compare mean standard errors: STAR with no softclipping *p* < 2.2e^−16^, R^2^ = 0.974, SSE = 8.73; STAR default: *p* < 2.2 × 10^−16^, R^2^ = 0.954, SSE = 16.22; Tophat2 with 5 mismatches: *p* < 2.2 × 10^−16^, R^2^ = 0.949, SSE = 17.57; Tophat2 default: *p* < 2.2 × 10^−16^, R^2^ = 0.851, SSE = 57.27) (Fig. 2b). To explore further whether the number of mismatches allowed influenced our ability to detect the correct proportion of reference alleles in different regions of the genome for each software, we repeated our analysis above and performed a parameter sweep from 1 to 10 mismatches allowed on average per read for Tophat2, STAR and STAR (with soft clipping disabled) using one of our simulated datasets. In stable regions of the genome (those with 2 or fewer heterozygous SNVs per MB) we find that generally there is a good match between the proportion of reference alleles detected across all heterozygous sites compared to the ground truth when 3 or more mismatches are allowed per read (R^2^ > 0.9 for all software, see Additional File 2: Figures S1, 2 and 3). For these regions our normalization approach had a similar performance to mapping reads without correction. For highly variable regions (those in the top 2.5 % of MBs, >30 heterozygous SNVs per MB), the correlation between mapped data and the ground truth for the proportion of reference alleles at heterozygous sites was much weaker (as low as R^2^ = 0.18 for Tophat2 allowing 1 mismatch per read, more generally R^2^ ~ 0.8 for 3 or more mismatches allowed per read), yet in this case our normalization approach greatly improved the strength of correlations (3 mismatches or greater, R^2^ ~ 0.9 for all software, see Additional File 2: Figures S1, 2 and 3).

Following this we considered how well ASE events were called in both the original mapping data for the four approaches outlined above, as well as after normalization in each case, by comparing calls to the underlying truth in simulated datasets. To do this we considered only heterozygous sites covered by at least 12 sequencing reads (overlapping pairs counted once) and used a binomial test with *P* < 0.005 to define a site as undergoing ASE. On average across each of the five simulated datasets, STAR with no soft clipping (sensitivity = 82.66 %, specificity = 99.56 %, precision = 74.61 %) and Tophat2 with 5 mismatches (sensitivity = 81.78 %, specificity = 99.57 %, precision = 74.72 %) performed best, followed by default STAR mapping (sensitivity = 71.40 %, specificity = 99.25, precision = 59.87 %) and then default Tophat2 mapping (sensitivity = 59.15 %, specificity = 96.14 %, precision = 19.32 %) (Table 1). Again, our normalization approach improved the precision of calling ASE events in all cases (Table 1). Overall, mapping with Tophat2 (5 mismatches) and applying our normalization method gave the best precision of 88.03 % (vs 83.50 % after normalization, mapping with STAR and no soft clipping). Applying our normalization approach to data mapped with STAR (with no soft clipping) resulted in the best sensitivity (82.91 %), although this was only marginally better than after normalising mapping data from Tophat2 (5 mismatches), which was 80.97 %.

**Table 1:** A comparison of ASE call rates for original mapped data and after normalization, for four different alignment methods

Method	True Positives	False Positives	True Negatives	False Negatives	Sensitivity	Specificity	Precision
Tophat2	108.2	452.4	11281.0	74.6	59.15 %	96.14 %	19.32 %
Tophat2 Normalized	110.4	94.0	11639.4	72.4	60.33 %	99.20 %	53.90 %
STAR	130.8	87.6	11666.8	52.4	71.40 %	99.25 %	58.87 %
STAR Normalized	135.2	40.2	11714.2	48.0	73.75 %	99.66 %	77.03 %
TH2_5MM	149.6	50.6	11702.8	33.4	81.78 %	99.57 %	74.72 %
TH2_5MM Normalized	148.2	20.0	11733.4	34.8	80.97 %	99.83 %	88.03 %
STAR (No clip)	151.6	51.4	11703.0	31.6	82.66 %	99.56 %	74.61 %
STAR (No clip) Normalized	152.0	30.0	11724.4	31.2	82.91 %	99.74 %	83.50 %

In all cases, the true number of significant ASE events averaged across five simulations is 183.2

Next, we generated an additional 20 simulated RNA sequencing datasets (making a total of 25) and compared our normalization method to both a site filtering approach and mapping to both parental genomes in order to detect ASE events. In each case we mapped with both Tophat2 (with 5 mismatches) and STAR (disabling soft-clipping), given that these approaches performed best during testing on a smaller number of simulated datasets (above). On average, each simulated dataset contained 179.4 genuine ASE events. Using STAR (no soft clipping) mapping and applying our normalization method, 147.4 true positives and 29.0 false positives were detected, leading to sensitivity of 82.28 %, specificity of 99.75 % and precision of 83.52 %. Mapping to both parental genomes performed marginally better overall with sensitivity, specificity and precision of 84.80 %, 99.81 % and 87.17 % respectively, whereas the filtering method performed worse (sensitivity = 68.53 %, specificity = 99.63 %, precision = 73.70 %) (Table 2). Mapping with Tophat2 (5 mismatches) and applying our normalization approach allowed for the detection of 142.28 true ASE events and 19.80 false positives, leading to sensitivity, specificity and precision of 79.42 %, 99.83 % and 87.69 % respectively. In this case, both the filtering method and mapping to two genomes had a lower precision (Table 2). Overall, mapping with Tophat2 (5 mismatches) and applying our normalization method leads to the best precision.

**Table 2:** A comparison of ASE call rates for three different approaches: a filtering methods, mapping to two parental genomes and our normalization approach

Mapping procedure	Control Method	True Positives	False Positives	True Negatives	False Negatives	Sensitivity	Specificity	Precision
TH2_5MM	Normalized	142.28	19.80	11737.80	36.80	79.42 %	99.83 %	87.69 %
TH2_5MM	Two Genomes	144.96	21.84	11737.68	34.44	80.84 %	99.81 %	86.85 %
TH2_5MM	Filtered	117.44	46.12	11711.48	61.64	65.71 %	99.61 %	71.77 %
STAR (No clip)	Normalized	147.40	29.00	11729.24	31.80	82.28 %	99.75 %	83.52 %
STAR (No clip)	Two Genomes	152.24	22.28	11724.96	27.16	84.80 %	99.81 %	87.17 %
STAR (No clip)	Filtered	122.68	43.72	11714.52	56.52	68.53 %	99.63 %	73.70 %

### RNA sequencing data

We sequenced the whole exomes and transcriptomes of 96 individuals from whole blood, called SNVs and indels from the exome and RNA data and mapped and filtered RNA sequencing reads with both Tophat2 and STAR, before applying our normalization method to detect ASE events (removing three outlier individuals, see methods). Final results obtained were quantitatively similar (Additional File 3 Table S2) for both mapping procedures, so from this point on we discuss the results obtained from Tophat2 mappings. After initial sequence alignment, each individual had an average of 9895 heterozygous sites covered by at least 12 sequencing reads (counting overlapping fragments only once). Of these, an average of 726.6 ASE events were identified per individual, representing 7.34 % of sites, although clearly this value is highly dependent on the coverage threshold used. After remapping the data to tolerate a higher number of mismatches (as above) and applying our normalization approach, 10017 sites were covered by at least 12 non-overlapping reads (reflecting the improved mapping procedure) and 550.1 of these sites were identified as undergoing ASE, which is consistent with the removal of false positives as highlighted during the analysis of simulated data.

To confirm the reliability of the data we hypothesised that adjacent heterozygous SNVs in each gene should have similar parental allelic proportions in genes predicted to have only one transcript (genes with multiple transcripts may be undergoing isoform specific expression [9]). Consistent with this we found that our normalization approach and altered mapping parameters significantly reduce allelic differences at adjacent sites compared to original mapped data (5.06 % on average, vs 5.89 % for original mapped data, *p* = 1.15 × 10^−57^). Furthermore, we found that our normalized dataset had an average reference allele proportion closer to the expected 50 % for 93 out of the 93 individuals, compared to original mapped data, and that the mean proportion of reference alleles at heterozygous sites was not significantly different from 50 % for 70 individuals after normalization (*p* < 0.05 after Bonferroni correction).

**Fig. 3:**
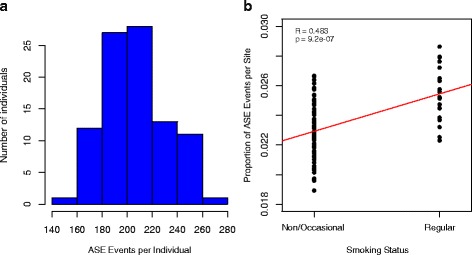
ASE calls per individual. The number of sites showing ASE per individual after resampling to depth 20X (**a**) and the relationship between the proportions of ASE events per site, per individual, and smoking status (**b**)

Finally, in order to consider the biological context of allele specific expression, we compared the proportion of sites undergoing ASE in each individual with five lifestyle traits (smoking, sun exposure, fruit and vegetable intake, levels of sleep and alcohol intake), adjusting for age, sample site and blood cell counts. Since it is more likely that ASE events are detectable at higher coverage [22], in order to compare across individuals, we resampled each heterozygous site without replacement to a depth of 20X, and relaxed our threshold for detecting ASE events to *P* < 0.05. On average, each individual had 8822 sites covered by at least 20 RNA sequencing reads and of these, 207.3 showed significant evidence of ASE after normalization (range 157–271, Fig. 3a). Comparing these values to lifestyle traits across individuals, we found no significant associations for four of the traits (*p* > 0.05 in all cases), however we observe that the proportion of sites undergoing ASE in whole blood data is significantly associated with smoking (full model *p* = 2.85 × 10^−4^, Bonferroni corrected = 1.4 × 10^−3^, correlation p-value = 9.2 × 10^−7^, Fig. 3b). It is worth noting that using the original mapped data the relationship between these two variables is not significant (*P* = 0.411), reinforcing the need for the correct treatment of data for ASE analysis when attempting to uncover biologically relevant signals.

## Conclusions

The use of high-throughput sequencing to detect ASE is becoming increasingly common. The technique allows for the identification of biologically relevant signals at the level of the individual, many of which may have important implications for our understanding of human disease. However, the mapping of short read sequences has inherent biases, and as data sets grow to unprecedented scales, it is becoming increasingly important to develop fast and reliable methods to identify genuine ASE events.

By applying a new approach for the normalization of RNA sequencing data to simulated data sets, we highlight two important results. First, selection and fine-tuning of sequencing alignment methods can drastically improve ASE analysis, reducing false positives and improving signal detection. The use of higher thresholds for mismatch tolerance, sensible read clipping and rigorous filtering of ambiguously placed reads leads to improved results. Second, our normalization approach can be used to further improve the detection of ASE. Our approach performs better than general site filtering techniques, which while simple and quick to implement, often do not take into account the personal nature of genomes and can also over-filter biologically relevant signals. Our approach also performs broadly similar to mapping to two parental genomes (with greater precision, but lower sensitivity), but with far less complex manipulation of data on a per sample basis. In our hands, once the original data is aligned, it can be normalized using SNVs and indels from exome sequencing data in ~120 min per sample with STAR and ~210 min with Tophat2 (including null data generation, mapping and filtering of null data and normalization, using a single node, with two 2.667GHz six-core processors, ~65 million read pairs), thus allowing this approach to be applied in large-scale sequencing studies.

Applying our approach to RNA sequencing data from 96 individuals we find evidence that suggests a low error rate in calling the ratio of parental alleles at heterozygous sites. First, we find consistency in parental allele biases within genes with single isoforms, suggesting consistent patterns of expression. Second, we find that the data conforms more readily to expectations that on average parental alleles should show a 50:50 ratio of expression. Finally, we identify a relationship between the total proportion of ASE events per individual and smoking using RNA sequencing data from whole blood. Although many previous studies have identified variation in gene expression profiles between smokers and non-smokers [23–26], we demonstrate here a non-specific, genome-wide influence of smoking on the biased expression of alleles at heterozygous sites in whole blood data. This serves as an example of how carefully treated analysis of RNA sequencing data for ASE detection can be used to identify biologically relevant signals.

## Methods

### Study population and phenotypic data

The study was approved by the Ethical Review Board Committee of Sainte-Justine Research Center and informed consent was obtained from all participants. 96 male individuals were drawn from the CARTaGENE biobank [27] for exome and RNA sequencing. Whole blood samples were collected in EDTA tubes (for DNA) and in Tempus tubes (Life Technologies) for RNA work. All samples were stored at -80C until processing. Lifestyle phenotypes were obtained from CARTaGENE’s self administered module. Alcohol intake was split into six groups based on frequency of intake (never, less than once per month, monthly, 2/3 per month, 2/3 per week, everyday/almost everyday), sun exposure into six groups (time spent in sun during summer between 11 am-4 pm on weekdays, groups ranged from <30mins per week to 5–6 h per week), fruit and vegetable intake into four categories, sleep was sorted by average number of hours slept per night and smoking level was sorted into two groups of current regular (daily) smokers and current non-smokers (never smoked, past smoker, occassional smoker).

### DNA and RNA preparation and sequencing

Full details of preparation and sequencing procedures have been previously documented [28, 29]. In brief, total RNA was extracted using the Tempus Spin RNA isolation kit (Life Technologies) and globin mRNA-depletion performed with the GLOBINclear-Human kit (Life Technologies). A 2100 Bioanalyzer instrument (Agilent) was used to check the quantity and integrity of RNA and all samples in our study had an RNA Integrity Number (RIN) > 7.5. Paired-end RNASeq libraries were constructed using the TruSeq RNA Sample Prep kit v2 (Illumina) and 500 ng of globin mRNA-depleted total RNA. Quality control and quantification of RNA sequencing libraries were performed prior to sequencing using Illumina’s recommended protocols. 100 bp paired-end RNA sequencing was performed on the Illumina HiSeq 2000 platform, using three samples per sequencing lane, at the Genome Quebec Innovation Centre, Montreal, Canada. Exome paired-end library construction was performed using the TruSeq Exome Enrichment and TruSeq DNA LT Sample Prep v2 kits (Illumina) before 100 bp paired-end sequencing using six samples per sequencing lane on the HiSeq 2000 platform.

### Alignment and variant calling

Exome sequencing read ends were trimmed for adaptor sequences and nucleotides that had a phred-quality score <20 and data were mapped to a reference genome (hg19, European Hapmap (CEU) major allele release) with bwa-aln using default parameters [30]. PCR duplicates were removed with Picard-tools, properly paired reads were kept with samtools v1.1 [31] and non-uniquely mapped reads filtered. Local realignment and base score recalibration was performed using GATK tools v3.2-2 [21]. On average, we achieved a coverage of ~40X. Single nucleotide variants (SNVs) and Indels were called with GATK tools by first using the haplotype caller per individual, then genotype calling across samples and finally VQRS filtering of low quality SNVs and Indels. Variants were then filtering to keep sites with coverage > =10 and those in Hardy-Weinburg equilibrium (*p* > 0.001). Any SNVs falling within indels were removed.

RNA sequencing reads for the same samples were trimmed as above and then mapped to a reference genome (hg19, European Hapmap (CEU) major allele release) with two commonly used aligners, Tophat2 [19] and STAR [20], using the Ensembl gene set (v75). For Tophat2 we mapped using both the default parameters and also allowing for five mismatches per read. For STAR we implemented two-stage mapping with the default parameters and also while disabling softclipping (using –alignEndsType EndtoEnd). Reads were then filtered as above. SNV and Indel calling were performed for RNA sequencing data using GATK tools v3.3. The –SplitNCigarReads option was used prior to base score recalibration, haplotype calling using variants from dbSNP v128 and merging of the 96 gVCF files. After calling, hard filtering was applied with parameters *–cluster* 3, *FS* > 30, *QD* < 2 and coverage > 10. After SNV and indel calling, one individual was removed as an outlier in terms of the number of heterozygous SNVs. Since all samples were from males, for all analyses we focus on the autosomes.

### Normalization method

After mapping and filtering we generated a high coverage “null” dataset for correction of the original RNA sequencing data using the following method. Using the mapped RNA sequencing data, read and read pairs covering the first heterozygous SNV site (as identified from exome sequencing) were identified using samtools (RNA SNV/Indel calls were also included for real data). Then, each read pair covering that site was extracted and copied, generating two identical synthetic read pairs in each case. One of these pairs was then scanned for the presence of heterozygous SNVs/Indels using exome sequencing calls – if a variant was found, the read was modified to contain the opposite parental allele. This was repeated for all variants within the read pair (tallying the number of SNVs modified for each pair and storing this as the *SNPweight* of the read pair), and then for all reads covering the heterozygous site, ultimately creating two sets of read pairs, each of which represented one of the two parental haplotypes. Sets of read pairs containing each parental haplotype were then grouped together and sampled randomly with replacement to generate coverage of 4000X. During this process, sets were kept with probability 1/*SNPweight* to ensure that even coverage was obtained across neighbouring heterozygous sites. The process was then repeated across all heterozygous SNVs, and reads were combined and converted to *fastq* format, thus generating a null dataset where parental haplotypes were present at exact 50:50 ratios across the entire transcriptome.

Null *Fastq* files were then remapped onto the genome using exactly the same approach as the original RNA sequencing data. In this way, since all alleles present at heterozygous sites were present at exact 50:50 ratios, any biases in the mapping process could be identified. After mapping of the null data, allele counts were obtained at each heterozygous site using a custom script, and used to normalize the original RNA sequencing data using the following formula:

$$ {A}_{ref}=\left(\frac{\left(Nul{l}_{ref}-{S}_{ref}\right)}{S_{cov}}\times {O}_{cov}\right)+{O}_{ref} $$

Where *A*_*ref*_ = adjusted reference allele count, *Null*_*ref*_ = number of reference alleles in null data, *S*_*ref*_ = observed reference allele count in null data, *O*_*ref*_ = observed reference allele count in real data, *S*_*cov*_ = sampled coverage at site in null data, *O*_*cov*_ = observed coverage at site in real data. *A*_*ref*_ represents the adjusted number of reference alleles after correction using the null data, which is then rounded to the nearest whole number to be compatible with a binomial test. A similar calculation was performed for the alternative allele, and then combined with the above to get the adjusted coverage.

### Simulations

In order to test the performance of our approach, we generated a simulated transcriptome that contained variants present in one of our sequenced samples, with parental alleles at known proportions that mirror the real data.

SNV and Indel calls generated from exome sequencing data were phased with ShapeIT2 [32], using read-aware phasing across the 96 samples. French-Canadian recombination maps were obtained from Hussin et al. [29] and data was split into chromosomes for phasing, except for chromosome 1, which was split into arms at the centromere. Trimmed RNA sequencing data for a single sample was mapped to a reference genome (hg19, European Hapmap (CEU) major allele release, Ensembl gene set) with RSEM [33], generating expression levels for each transcript. The option *rsem-simulate-reads* was then used to generate simulated RNA sequencing data, with input parameters generated from the original mapping results. Since the simulated dataset contained no SNVs or Indels, we used a custom script to insert phased variants obtained from exome sequencing data for the same individual into the simulated sequencing reads. During this step, to generate a range of parental allele ratios to insert into simulated data we calculated the proportions of reference alleles at each heterozygous site in the real RNA sequencing data for the same individual (where coverage was >8X), which was then centred at 50 % (by adding 1-*x* to the distribution for each site, where *x* is the proportion of reference alleles at that heterozygous site. For the final reference allele distribution, see Additional File 2: Figure S4). Each gene was then randomly assigned a value from this distribution, and any heterozygous SNVs/Indels falling within that gene were inserted with the corresponding reference allele proportion. This process was repeated multiple times, generating many independent simulated datasets where the ratio of reference to alternative alleles at each heterozygous site was known (the ground truth) and could be compared to mapped and normalized data. Alternative distributions were also generated and tested, and in each case the general results were similar to those reported in the main text (see Additional File 2: Figure S4 and Additional File 3: Table S1).

### Method comparisons

We compared our normalization approach to two commonly used methods: the filtering approach and mapping to two parental genomes. For the filtering approach we removed heterozygous sites that fell into low mappability regions (CRG map score < 1), those that have known biases using 1000 Genomes data when mapping with either BWA or GEM [34] and sites that are in known duplicated regions [35]. Following this, we recalculated the average reference allele proportion using the remaining sites, and used this as the expected value within a binomial test under the same criteria as outlined above. For the two parental genomes approach we took the original SNV and Indel calls generated from exome sequencing data for the individual in question and phased them with ShapeIT2 using the 1000G reference panel. SNVs and Indels not on the reference panel were assigned randomly to one of the two haplotypes. Parental genomes for the simulated individual were generated using the vcf2diploid program within AlleleSeq [18] and parental GTF files were created using the output files and liftOver tool. We then mapped the simulated data to both parental genomes using the same mapping tools and filtering pipelines as outlined above for the original data. Read pairs mapping uniquely to either of the parental genomes were kept and the best match was found for any read pairs mapping to both genomes, scoring the read pairs with +1 per nucleotide match and −2 per indel mismatch. In all cases, if significant ASE occurs at a site in the ground truth data, true positives are counted if the method also shows significant ASE, with the allelic bias occuring in the same direction as the ground truth.

### Allele specific expression and phenotype data

Heterozygous sites were tested for ASE using the binomial test with *p* < 0.005 if they had coverage > =12X and both alleles were found to be present in RNA sequencing data. In all cases, we consider overlapping segments of read pairs only once using the software bamUtil and allele counts were obtained using samtools 1.1, with no BAQ correction and overlaps ignored. For real RNA sequencing data, one individual was initially removed as an outlier for the number of heterozygous sites covered by at least 20 non-overlapping sequencing reads (Additional File 2: Figure S5).

To compare allele proportions for adjacent heterozygous SNVs in genes with a single documented transcript (as obtained from the Ensembl gene set v75), on a per individual basis we collected heterozygous SNVs within each gene, subtracted the reference allele proportion from one if it was above 50 % (to account for unknown phase), and then calculated the absolute difference between each adjacent SNVs. To generate a null distribution, random pairs were drawn from the same set of heterozygous SNVs and compared as above.

To consider the relationship between ASE and lifestyle traits, ASE events were identified by resampled each heterozygous site without replacement to a depth of 20X and then performing binomial tests with a relaxed p-value (*P* < 0.05). The proportion of sites undergoing ASE was then calculated by dividing the number of ASE events by the number of heterozygous SNVs that had at least 20X coverage on a per individual basis, thus controlling for the effects of sequence depth variation. Two individuals were outliers for the proportion of sites undergoing ASE (Additional File 2: Figure S6) and were removed from all analysis.

## Additional files

Additional file 1: Pipelines and examples to implement the ASE normalization software. (PDF 111 kb) Additional file 2: Supplementary Figures and Tables. (DOCX 978 kb) Additional file 3:
**Table S2** detailing the numbers of covered sites and ASE events within RNA sequencing data from 93 individuals. (XLSX 45 kb)

## Abbreviations

ASE: Allele specific expression
SNV: Single nucleotide variants (SNVs)
SSE: Sum of squared errors
