# Supplementary material for: A haplotype-based normalization technique for the analysis and detection of allele specific expression
Source: BMC Bioinformatics. 2016 Sep 13;17(1):364. doi: 10.1186/s12859-016-1238-8 (PMC5020486; doi:10.1186/s12859-016-1238-8)
Supplement: Additional file 1: — Pipelines and examples to implement the ASE normalization software. (PDF 111 kb) [file 12859_2016_1238_MOESM1_ESM.pdf]

Allele Specific Expression Normalization

This tool is designed to provide a method by which aligned RNA sequencing data can be normalized in order to more accurately detect allele specific expression. Since normal sequencing mapping procedures often result in biases, particularly around indels and highly polymorphic regions, the method works by considering whether each genetic variant would be mapped correctly when both parental alleles are present at a 50:50 ratio within a simulated 'null' dataset. Biases that are detected in the null dataset are then used to correct the original data.

The following outlines a basic pipeline for implementing the software, including all software options, followed by an example using test data. All software and example datasets are available at https://github.com/AJHodgkinson/ASE.

Pipeline for implementing the software

The following describes the step by step process required to use the normalisation software.

Programs: ase_normalisation_sim.pl and ase_normalisation_compare.pl

These two programs can be used to normalize mapped Paired End RNA sequencing files (bam) and produce an output file giving a P-value for the likelihood that the site is deviating from an allele ratio of 50:50.

Step 1: Map RNA Sequencing Data with software of choice

Examples:

```
STAR --genomeDir STARgenome2pass --runThreadN threads --alignEndsType
EndToEnd --readFilesIn fastq_1.fq.gz fastq_2.fq.gz --readFilesCommand
zcat --outFileNamePrefix Outname --outFilterMismatchNmax 10
```

```
or
```

```
tophat2 -o Outname -N 5 --read-edit-dist 5 -p threads
--transcriptome-index=index bowtie2_index fastq_1.fq.gz fastq_2.fq.gz
```

This can then be followed by desired filtering strategies such as PCR duplicate removal and/or keeping properly paired and uniquely mapped reads. Note that in order to perform the next step, only paired reads should be kept (non-paired reads will be discarded and may thus bias the simulation).

Step 2: Use the mapped data as the basis to create the simulated dataset:

```
perl ase_normalisation_sim.pl [options] --DNAvcf <VCF_file_name>
--DNAid <ind_id_from_vcf> --OUTid <Outfolder_name> --RNAbam
<Mapped_RNA_BAM>
```

Where --DNAvcf is the name and path to VCF file containing variation that will be used within the simulation, --DNAid is the name of the individual as shown in the VCF file, --OUTid is the name of output folder (also used in output file names - directory will be created with this name and simulation files will be produced within this directory, --RNAbam is the name of the BAM file containing mapped RNA sequencing data for the individual of interest (This file must contain properly paired, uniquely mapped reads), --MaxProcs allows the simulation to be run in parallel on multiple cpus (we recommend using no more than 8 cpus on a node containing 12 cpus, default: 1), and --SampleDepth is the depth of reads to be simulated across each site (increasing this may improve accuracy during normalisation, decreasing will improve speed of simulation and subsequent mapping, default: 2000).

Step 3: Map the simulated null dataset:

The simulation program will generate two fastq.gz files containing read pairs. Map these files using the exact same approach that you used in step 1. The simulation program will also produce two count files showing the number of reference (and thus alternative) reads covering each site for both trimmed (ignoring read pair overlaps) and non-trimmed data. These are for use in the ase_normalisation_compare.pl program to generate results.

Step 4: Correct the original data using the null dataset:

Use the original mapped data, the simulated mapped data and the count files (Null_sim_file below) to generate P-values for each heterozygous site:

```
perl ase_normalisation_compare.pl [options] --DNAvcf <VCF_file_name> --DNAid <ind_id_from_vcf> --OUTid <Outfolder_name> --RNAbam <Mapped_RNA_BAM> --RNASIMbam <Mapped_RNASIM_BAM> --Ref <Reference_fasta> --MapNull <Null_sim_file>
```

Where --DNAvcf is the name and path to VCF file containing variation that will be used within the simulation, --DNAid is the name of the individual as shown in the VCF file, --OUTid is the name of Output folder (also used in output file names - directory will be created with this name and simulation files will be produced within this directory), --RNAbam is the name of the BAM file containing mapped RNA sequencing data for the individual of interest, --RNASIMbam is the name of the BAM file containing mapped RNA sequencing data for the individual of interest, generated from the simulation program, --Ref is the full path to Reference fasta file for use in Samtools Mpileup (this should be the same reference as used to call SNVs present in the supplied VCF file), --MapNull is the path to null allele file generated by the simulation program, --MaxProcs allows the simulation to be run in parallel on multiple cpus (we recommend using no more than 8 cpus on a node containing 12 cpus, default: 1) and --Baq is used to turn on probabilistic realignment for the computation of base alignment quality in Samtools Mpileup (default: off).

The results file will detail allele counts before and after normalisation, as well as a P-value from a binomial test.

Software Flags

The following is the full list of options available for the ase_normalisation_sim.pl program:

--DNAvcf <VCF_file_name> :	*Name and path to VCF file containing variation that will be used within the simulation. (Required)*
--DNAid <ind_id_from_vcf> :	*Name of the individual as shown in the VCF file. (Required)*
--OUTid <Outfolder_name> :	*Name of Output folder (also used in output file names). Directory will be created with this name and simulation files will be produced within this directory. (Required)*
--RNAbam <Mapped_RNA_BAM> :	*Name of the BAM file containing mapped RNA sequencing data for the individual of interest. This file must contain properly paired, uniquely mapped reads. (Required)*
--MaxProcs <number_of_cpus> :	*The simulation can be run in parallel on multiple cpus. We recommend using no more than 8 cpus on a node containing 12 cpus. [default: 1] (Optional)*
--SampleDepth <depth_of_reads> :	*The depth of reads to be simulated across each site. Increasing this may improve accuracy during normalisation, decreasing will improve speed of simulation and subsequent mapping. [default: 2000] (Optional)*
--ADDvcf <additional_vcf> :	*It is possible to supply an additional VCF file, and additional variation not present in the required VCF will be simulated in surrounding regions. This variation will not be used for read collection at heterozygous sites, but will be used if it falls within selected reads. One example would be to supply a VCF from exome sequencing in order to find heterozygous SNVs and simulate reads across them, and then provide a whole-genome VCF to add extra SNPs around these sites for more realistic simulations. (Optional)*
--ADDid <id_additional_vcf> :	*If an additional VCF file is provided, use this option to define the name of the individual as specified in that VCF (can be different from ID in required VCF file). (Optional)*

The following is the full list of options available for the ase_normalisation_compare.pl program:

--DNAvcf <VCF_file_name> :	*Name and path to VCF file containing variation that will be used within the simulation. (Required)*
--DNAid <ind_id_from_vcf> :	*Name of the individual as shown in the VCF file. (Required)*
--OUTid <Outfolder_name> :	*Name of Output folder (also used in output file names). Directory will be created with this name and simulation files will be produced within this directory. (Required)*
--RNAbam <Mapped_RNA_BAM>:	*Name of the BAM file containing mapped RNA sequencing data for the individual of interest. (Required)*
--RNASIMbam <Mapped_RNA_BAM>:	*Name of the BAM file containing mapped RNA sequencing data for the individual of interest, generated from the simulation program. (Required)*
--Ref <Reference_fasta>:	*Full path to Reference fasta file for use in Samtools Mpileup. This should be the same reference as used to call SNVs present in the supplied VCF file. (Required)*
--MapNull <Null_sim_file> :	*The path to null allele file generated by the simulation program. (Required)*
--MaxProcs <number_of_cpus> :	*The simulation can be run in parallel on multiple cpus. We recommend using no more than 8 cpus on a node containing 12 cpus. [default: 1] (Optional)*
--Baq :	*Use to turn on probabilistic realignment for the computation of base alignment quality in Samtools Mpileup (BAQ). [default: off] (Optional)*

Example Pipeline and Data

As an example we have provided fastq files, bam files and an example results file at https://github.com/AJHodgkinson/ASE that can be used to test the software. The following details the steps that can be taken in order to reproduce the results in the example file:

The following files have been provided at https://github.com/AJHodgkinson/ASE:

- Sample fastq files: OUT_RNASeq_PE_sim_ind1_SNPs_1_chr22.fq and OUT_RNASeq_PE_sim_ind1_SNPs_2_chr22.fq - This data was generated by a custom script and is loosely based on the NA12812 1000G individual. It contains simulated data for a single chromosome.
- Mapped data: ind1_snps_chr22.Aligned.out.sort.PP.UM.bam - The above files were mapped with STAR and filtered. If you don't want to map the fastq files yourself, this bam file can be used in the simulation program.
- SNP file: ind1.vcf - this file shows the locations of polymorphisms in the fastq files and is to be used in the simulation program.
- A results file: all_results_ind1_1_STAR_gatk.txt - Having used the normalisation software on the above files, your results should be similar to those in this file.

To recreate the results file:

1) Generate a high coverage null dataset with the files provided by running the following command (assuming access to at least 12 cpus, otherwise change to --MaxProcs 1):

```
perl ase_normalisation_sim.pl --DNAid ind1 --OUTid ind1 --
DNAvcf ind1.vcf --RNAbam
ind1_snps_chr22.Aligned.out.sort.PP.UM.bam --MaxProcs 8 --
SampleDepth 2000
```

   This will produce a null allele file 'ref_allele_count_ind1.txt' for use in the final step

2) Map and filter the null dataset in the same way as the original data. If using the test BAM file, use the following approach:

```
#Map data (you will need to create a reference STAR genome with
hg19 reference):

STAR --genomeDir hg19 --readFilesIn fastq_sim_ind1_R1.fastq.gz
fastq_sim_ind1_R2.fastq.gz --runThreadN 12 --readFilesCommand
zcat --outSAMstrandField intronMotif --outFileNamePrefix
ind1_SNPs_chr22_deepsim.

#Covert SAM->BAM
samtools view -bh ind1_SNPs_chr22_deepsim.Aligned.out.sam >
ind1_SNPs_chr22_deepsim.Aligned.out.bam
```

```
#Sort
samtools sort ind1_SNPs_chr22_deepsim.Aligned.out.bam
ind1_SNPs_chr22_deepsim.Aligned.out.sort

#Index
samtools index ind1_SNPs_chr22_deepsim.Aligned.out.sort.bam

#Keep properly paired reads
samtools view -@ 12 -f 0x0002 -b -o
ind1_SNPs_chr22_deepsim.Aligned.out.sort.PP.bam
ind1_SNPs_chr22_deepsim.Aligned.out.sort.bam
samtools index ind1_SNPs_chr22_deepsim.Aligned.out.sort.PP.bam

#Keep Unique hits
samtools view -h
ind1_SNPs_chr22_deepsim.Aligned.out.sort.PP.bam | grep -P
"NH:i:1\t|^@" | samtools view -bS - >
ind1_SNPs_chr22_deepsim.Aligned.out.sort.PP.UM.bam
samtools index
ind1_SNPs_chr22_deepsim.Aligned.out.sort.PP.UM.bam
```

3) Use the compare software to get the allele counts before and after normalisation (use the hg19 fasta file used to create STAR reference above):

```
perl ase_normalisation_compare.pl --DNAid ind1 --OUTid ind1 --
DNAvcf ind1.vcf --RNAbam
ind1_snps_chr22.Aligned.out.sort.PP.UM.bam --RNASIMbam
ind1_SNPs_chr22_deepsim.Aligned.out.sort.PP.UM.bam --Ref
hg19.fasta --MapNull ref_allele_count_ind1.txt --MaxProcs 8
```

Although the results file you generate may not be exactly the same as the results file provided (as the software relies on random sampling of reads), the normalized read counts should correlate strongly across the two results files.
