# Supplementary material for: A haplotype-based normalization technique for the analysis and detection of allele specific expression
Source: BMC Bioinformatics. 2016 Sep 13;17(1):364. doi: 10.1186/s12859-016-1238-8 (PMC5020486; doi:10.1186/s12859-016-1238-8)
Supplement: Additional file 2: — Supplementary Figures and Tables. (DOCX 978 kb) [file 12859_2016_1238_MOESM2_ESM.docx]

Supplementary material for:

**Title: A haplotype-based normalization technique for the analysis and detection of Allele Specific Expression**

**Authors:** Alan Hodgkinson^1,3,*^, Jean-Christophe Grenier^1^, Elias Gbeha^1,2,4^ and Philip Awadalla^1,2,4^

**Affiliations:**

1. CHU Sainte Justine Research Centre, Department of Pediatrics, Faculty of Medicine, Universite de Montreal, 3175 Chemin de la Cote Sainte Catherine, Montreal, Quebec, Canada

2. Ontario Institute of Cancer Research, Toronto, Ontario, Canada

3. Department of Medical and Molecular Genetics, Guy's Hospital, King’s College London, London, SE1 9RT, UK

4. Department of Molecular Genetics, University of Toronto, Toronto, Ontario, Canada

**Email:**

Alan Hodgkinson: alan.j.hodgkinson@gmail.com

Jean-Christophe Grenier: jean.christophe.grenier@gmail.com

Elias Gbeha: elias.gbeha@gmail.com

Philip Awadalla: philip.awadalla@umontreal.ca

* corresponding author: Alan Hodgkinson, alan.j.hodgkinson@gmail.com

**Figure S1**: Correlations between the proportion of reference alleles at heterozygous sites in mapped or normalized data versus the ground truth for Tophat 2. A simulated data set was used where the underlying proportions of refence alleles were known. Stable regions are thise with 2 or fewer heterozygous SNVs per MB and variable regions are thos with greater than or equal to 30 heterozygous SNVs per MB. The number of mismatches allowed per read was varied between 1 and 10.

**Figure S2**: Correlations between the proportion of reference alleles at heterozygous sites in mapped or normalized data versus the ground truth for STAR. A simulated data set was used where the underlying proportions of refence alleles were known. Stable regions are thise with 2 or fewer heterozygous SNVs per MB and variable regions are thos with greater than or equal to 30 heterozygous SNVs per MB. The number of mismatches allowed per read was varied between 1 and 10 (for STAR the number of mismatches per read pair is used, so the numbers above were doubled during the mapping procedure).

**Figure S3**: Correlations between the proportion of reference alleles at heterozygous sites in mapped or normalized data versus the ground truth for STAR (no soft clipping). A simulated data set was used where the underlying proportions of refence alleles were known. Stable regions are thise with 2 or fewer heterozygous SNVs per MB and variable regions are thos with greater than or equal to 30 heterozygous SNVs per MB. The number of mismatches allowed per read was varied between 1 and 10 (for STAR the number of mismatches per read pair is used, so the numbers above were doubled during the mapping procedure).

**Figure S4**: Reference allele proportions used in simulation studies. The distribution used for results in the main text is shown in the top left hand panel. The top right panel shows a distribution with 20% of the values between 45-55% removed, thus increasing the proportion of sites falling towards the tails of the distribution, and the bottom left hand panel shows 40% of values removed from the 45-55% band. Finally, the bottom right panel shows the original distribution that is not corrected around 50% and so has a reference allele bias.

**Figure S5**: The number of heterozygous sites covered by at least 20 non-overlapping sequencing reads. The outlier on the right hand side of the plot was removed from subsequent analysis.

**Figure S6**: ASE calls per individual. The proportion of sites showing ASE per individual after resampling to depth 20X. The two individuals on the right hand side of the plot were removed as outliers.

**Table S1**: A comparison of ASE call rates for original mapped data and after normalisation, for four different alignment methods using three different underlying distributions of reference allele proportions.

| **Method** | **ASE Events** | **True Positives** | **False Positives** | **True Negatives** | **False Negatives** | **Sensitivity** | **Specificity** | **Precision** |
| --- | --- | --- | --- | --- | --- | --- | --- | --- |
| **Distribution: 20% removed from 45-55% band:** | | | | | | | | |
| Tophat2 | 204.6 | 166.4 | 51.2 | 11681.6 | 37.8 | 81.54% | 99.56% | 76.41% |
| Tophat2 Normalised | 204.6 | 166.6 | 25.4 | 11707.4 | 37.6 | 81.60% | 99.78% | 86.66% |
| STAR | 204.6 | 149.0 | 89.8 | 11643.2 | 55.2 | 73.16% | 99.23% | 62.33% |
| STAR Normalised | 204.6 | 154.4 | 35.2 | 11697.8 | 49.8 | 75.68% | 99.70% | 81.33% |
| TH2_5MM | 204.6 | 164.0 | 55.6 | 11677.4 | 40.2 | 80.24% | 99.53% | 74.53% |
| TH2_5MM Normalised | 204.6 | 161.4 | 24.2 | 11708.8 | 42.8 | 78.97% | 99.79% | 86.86% |
| STAR (No clip) | 204.6 | 122.8 | 468.4 | 11246.6 | 81.0 | 60.36% | 96.00% | 20.77% |
| STAR (No clip) Normalised | 204.6 | 120.2 | 89.8 | 11625.2 | 83.6 | 58.97% | 99.23% | 57.12% |
| **Distribution: 40% removed from 45-55% band:** | | | | | | | | |
| Tophat2 | 205.0 | 173.4 | 54.2 | 11678.6 | 31.4 | 84.76% | 99.54% | 76.06% |
| Tophat2 Normalised | 205.0 | 172.8 | 31.2 | 11701.6 | 32.0 | 84.48% | 99.73% | 84.58% |
| STAR | 205.0 | 152.0 | 85.0 | 11647.6 | 52.8 | 74.23% | 99.28% | 64.16% |
| STAR Normalised | 205.0 | 157.6 | 39.2 | 11693.4 | 47.2 | 77.06% | 99.67% | 79.98% |
| TH2_5MM | 205.0 | 167.4 | 55.8 | 11675.4 | 37.2 | 81.82% | 99.52% | 74.89% |
| TH2_5MM Normalised | 205.0 | 166.0 | 26.0 | 11705.2 | 38.6 | 81.16% | 99.78% | 86.39% |
| STAR (No clip) | 205.0 | 123.0 | 465.4 | 11246.6 | 81.0 | 60.37% | 96.03% | 20.87% |
| STAR (No clip) Normalised | 205.0 | 124.8 | 93.4 | 11618.6 | 79.2 | 61.30% | 99.20% | 57.17% |
| **Distribution: original distribution, biased towards reference allele:** | | | | | | | | |
| Tophat2 | 203.2 | 162.2 | 50.4 | 11683.8 | 40.6 | 80.06% | 99.57% | 76.30% |
| Tophat2 Normalised | 203.2 | 166.2 | 29.0 | 11705.2 | 36.6 | 82.05% | 99.75% | 85.15% |
| STAR | 203.2 | 142.2 | 95.4 | 11638.8 | 60.6 | 70.11% | 99.19% | 59.90% |
| STAR Normalised | 203.2 | 147.8 | 40.6 | 11693.6 | 55.0 | 72.98% | 99.65% | 78.49% |
| TH2_5MM | 203.2 | 163.2 | 60.6 | 11673.4 | 39.6 | 80.63% | 99.48% | 73.05% |
| TH2_5MM Normalised | 203.2 | 162.2 | 26.6 | 11707.4 | 40.6 | 80.12% | 99.77% | 86.09% |
| STAR (No clip) | 203.2 | 119.2 | 475.4 | 11239.4 | 83.4 | 58.81% | 95.94% | 20.04% |
| STAR (No clip) Normalised | 203.2 | 119.4 | 90.6 | 11624.2 | 83.2 | 58.96% | 99.23% | 56.90% |
